# Recurrent carotid sinus syncope in a middle-aged woman without any underlying condition: a case report

**DOI:** 10.1097/MS9.0000000000003479

**Published:** 2025-06-13

**Authors:** Abdulrahman Saad Alfaiz

**Affiliations:** Department of Internal Medicine, Shaqra University, Riyadh, Saudi Arabia

**Keywords:** carotid sinus hypersensitivity, carotid sinus massage, carotid sinus syndrome, Fludrocortisone, syncope, vasodepressor

## Abstract

**Introduction and importance:**

Syncope is a frequent reason for emergency room visits, often triggering extensive diagnostic evaluations. However, a well-conducted bedside history and physical examination can significantly streamline the process, leading to an accurate diagnosis and reducing unnecessary testing. Carotid sinus syndrome (CSS), though relatively rare, is an important consideration – particularly in middle-aged and older adults.

**Case presentation:**

We report the case of a 53-year-old woman who had been experiencing recurrent episodes of syncope for 13 years. Despite undergoing multiple investigations, no definitive diagnosis had been established. However, a thorough clinical examination, combined with carotid sinus massage (CSM), ultimately identified CSS with a vasodepressor response. She was advised to adopt lifestyle modifications, including avoiding known triggers, increasing fluid intake, and taking Fludrocortisone 0.1 mg daily. A 1-year follow-up revealed complete resolution of her syncopal episodes.

**Clinical discussion:**

CSS is often an overlooked cause of recurrent syncope, requiring specific diagnostic techniques such as CSM for identification. The vasodepressor variant, in particular, is best managed with conservative strategies, patient education, and volume expansion. While randomized controlled trials are lacking, anecdotal evidence supports the use of Fludrocortisone and midodrine in selected cases. This case underscores the critical role of history-taking and bedside assessment in the accurate diagnosis and effective management of reflex syncope.

**Conclusion:**

A meticulous clinical history and targeted physical examination remain essential in identifying rare causes of syncope, such as CSS. This approach not only ensures appropriate management but also helps prevent misdiagnosis, optimizes the use of healthcare resources, and ultimately improves patient outcomes.

## Introduction

Syncope is a common and important clinical presentation in the emergency department, often leading to traumatic injury, anxiety, and a significant reduction in quality of life. It can be both debilitating and costly, particularly in older adults^[^1^]^. Syncope can be categorized based on its underlying mechanisms, which include reflex-mediated syncope, cardiac syncope, and neurologic syncope. Reflex syncope, such as carotid sinus syndrome (CSS), occurs when the autonomic nervous system excessively responds to triggers like head turning or tight collars, resulting in a drop in blood pressure (BP) and heart rate. Cardiac syncope, which is more dangerous, is caused by arrhythmias or structural heart issues and often necessitates interventions such as pacemakers or implantable cardioverter defibrillators. Neurologic syncope, resulting from transient ischemic attacks (TIAs) or seizures, can be difficult to differentiate from reflex syncope, further complicating the diagnosis^[^2^]^.

**HIGHLIGHTS**

- A middle-aged woman with recurrent syncope for over a decade was diagnosed with carotid sinus syndrome (CSS) of the vasodepressor type.
- Despite extensive diagnostic workups, including Holter monitoring, magnetic resonance imaging, electroencephalogram, and vestibular testing, the diagnosis remained elusive until carotid sinus massage confirmed CSS.
- Emphasizes the importance of thorough history-taking and targeted physical examination in diagnosing syncope, reducing unnecessary investigations.
- The patient was managed with lifestyle modifications, education on avoiding triggers, increased fluid intake, and Fludrocortisone therapy, leading to symptom resolution over 1 year.
- Highlights the need to consider CSS in cases of unexplained syncope, particularly in atypical age groups, for cost-effective diagnosis and management.

Syncope occurs most frequently in two age groups: between ages 10 and 20 and between 60 and 80 years. In about 37% of patients, the underlying cause remains unidentified, highlighting the complexity of its diagnosis^[^3^]^. Among known causes, reflex syncope is the most frequently reported, accounting for 21% of cases, while cardiac syncope and orthostatic hypotension are less common, accounting for 9% each^[^4^]^. Reflex syncope is more commonly observed in women than men, especially after age 60, with a prevalence of 40% in women compared to 28% in men^[^5,6^]^. Carotid sinus hypersensitivity (CSH), a form of reflex syncope, is more prevalent in older adults and can lead to recurrent episodes of syncope. When syncope occurs as a result of CSH, it is termed CSS, which is an often under-recognized cause of recurrent syncope^[^7^]^.

The challenge in diagnosing syncope arises from the overlap of symptoms and the fact that common diagnostic tests often yield normal findings. Tests like electrocardiogram (ECG), magnetic resonance imaging (MRI), Holter monitoring, and electroencephalogram (EEG) are frequently employed but may not reveal the underlying cause, especially in cases of reflex syncope where these tests are inconclusive. This diagnostic difficulty is compounded by the fact that syncope in older adults is frequently multifactorial, with cardiovascular disease, diabetes, and neurological disorders contributing to the complexity^[^8^]^. Therefore, an in-depth understanding of the patient’s clinical history, combined with advanced diagnostic tools, is essential for accurate diagnosis and management.

The primary risk factors for CSS include advancing age, cardiovascular disease, head or neck trauma, and activities that stimulate the carotid sinus, such as shaving, tight collars, or head-turning^[^7,9^]^. Despite extensive testing, many cases of syncope remain unexplained, highlighting the need for specialized diagnostic procedures. Common diagnostic methods include Holter monitoring, ECG, MRI, and carotid sinus massage (CSM)^[^10^]^. CSM is particularly valuable in diagnosing CSS as it reproduces the symptoms of syncope and helps differentiate between the vasodepressor and cardioinhibitory subtypes^[^11^]^.

CSM involves applying gentle pressure to the carotid sinus, located between the sternocleidomastoid muscle and cricoid cartilage while monitoring heart rate and BP. This procedure helps diagnose vasodepressor-type CSS by evaluating the response of carotid baroreceptors to pressure. A positive result is indicated by a significant drop in BP or heart rate (greater than 50 mm Hg or bradycardia), which can induce syncope or near-syncope. The test is typically performed with the patient in a supine position, and care must be taken in patients with stroke, TIA, or carotid bruit due to potential risks. Vasodepressor-type CSS is typically managed conservatively with lifestyle changes and Fludrocortisone therapy, while the cardioinhibitory subtype may require more invasive treatments, such as pacemaker implantation^[^7,12^]^.

Vestibular testing plays an important role in ruling out neurologic causes of syncope, such as vestibular disorders. Caloric and rotary chair tests assess inner ear function, helping to differentiate between vertigo and reflex syncope^[^13^]^. Normal vestibular testing results suggest that syncope is more likely caused by a reflex-mediated mechanism like CSS rather than a vestibular or neurological issue. Vestibular testing is a valuable tool in the differential diagnosis of unexplained syncope and dizziness.

Prior studies have demonstrated the utility of vestibular testing in ruling out vestibular disorders, particularly in distinguishing them from reflex syncope. However, its role in diagnosing CSS remains underappreciated, as many studies focus more on cardiac and neurological causes of syncope. This case highlights the presentation of a middle-aged woman who experienced multiple episodes of syncope over a decade. Despite extensive investigations, her diagnosis remained elusive until she was eventually diagnosed with vasodepressor-type CSS, which was successfully managed through lifestyle modifications and Fludrocortisone therapy.

## Case presentation

### History and examination and past medical history

A 53-year-old female presented after a syncopal episode. It was characterized by a sudden occurrence of loss of consciousness, leading to a fall. The episode lasted for a few seconds, with the patient regaining full consciousness afterward. There were no prodromal symptoms. The episode was not followed by any confusion. No bowel or bladder incontinence, no tongue bite, and jerky movements. Upon further questioning, the patient reported that this was not the first time, and she has been having similar episodes for the last 13 years when she was 40-year-old. All those episodes occurred during walking, standing, or carrying out some physical activity and none could be recalled during resting or lying, however, there were no known triggers.

Although distressing, fortunately, none of those syncopal episodes had caused any major physical injuries. There was no history of diabetes mellitus, ischemic heart disease, valvular heart diseases, intracardiac masses, cardiopulmonary diseases, spinal cord injury, Parkinsonism, cerebrovascular disease, epilepsy, chronic kidney disease, metabolic syndromes, autoimmune diseases or brain or spinal cord tumors.

The patient’s past surgical history includes hernia repair, surgical excision of Bartholin cyst, and fibroadenoma breast. Her family history is unremarkable. She had many consultations with healthcare physicians regarding her condition and had been thoroughly investigated, but her condition remained a diagnostic dilemma.

#### Follow-up criteria

The patient’s improvement was assessed by a decrease in the frequency of syncopal episodes (from monthly to none within a year), her subjective report of symptoms (i.e. absence of dizziness or lightheadedness), and normal vital signs. These measures were tracked during regular follow-up visits at 1, 3, 6, and 12 months post-intervention.

**Figure 1.**
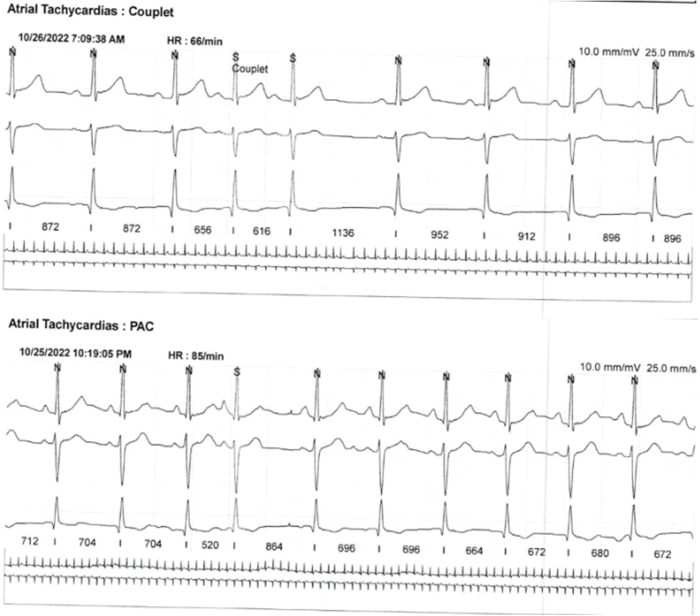
Rhythm strips from the patient’s Holter monitoring.

As part of the diagnostic workup, she had previously had Holter monitoring which showed sinus rhythm and infrequent ventricular ectopic beats as shown in Figure 1.

The upper strip shows couplets of atrial tachycardia with a heart rate of 66 beats per minute. These couplets represent two consecutive atrial premature beats (premature atrial complex [PAC]) followed by normal sinus rhythm. The couplet is a characteristic feature of atrial tachycardia, which can be a sign of atrial arrhythmias. The lower strip displays atrial tachycardia with PACs, occurring at a heart rate of 85 beats per minute. PACs are early beats originating from the atria, often seen in patients with atrial arrhythmias. Both strips demonstrate abnormal atrial activity captured during the patient’s Holter monitoring over different periods (October 2022). The findings indicate atrial arrhythmias, which may contribute to the patient’s syncopal episodes.

She also previously had MRI brain, MRI spine, and EEG and they all revealed nonspecific findings. Eventually, she underwent vestibular testing as well which was normal. The findings of her vestibular testing are shown in Figure 2ab,c.

**Figure 2.**
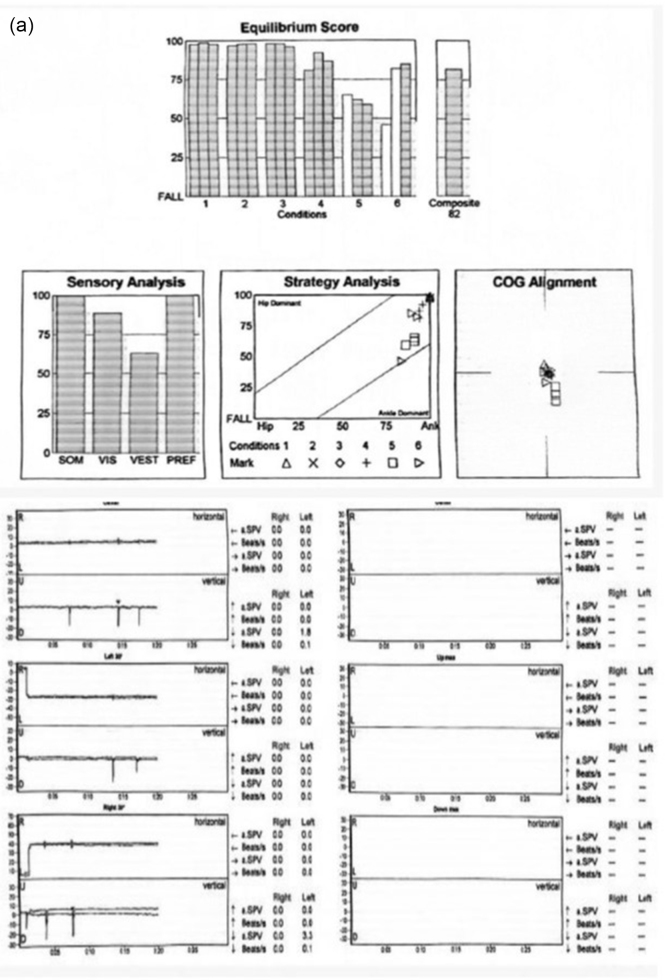

**Figure 2.**
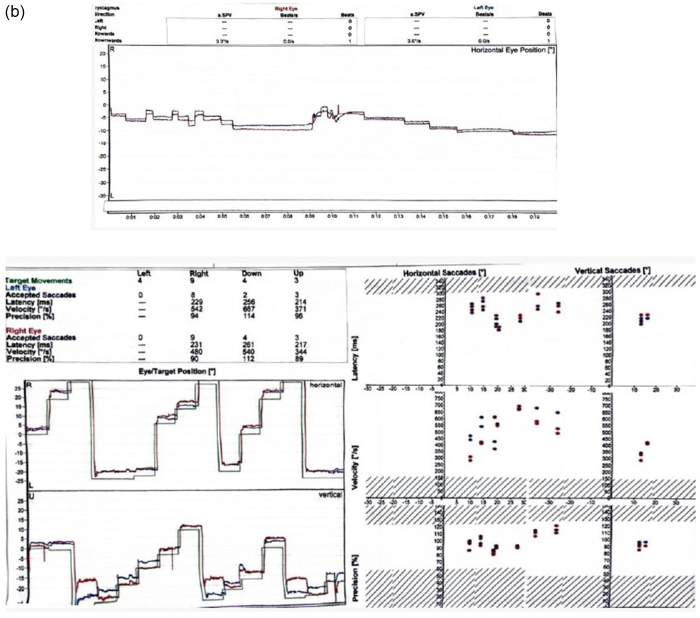

**Figure 2.**
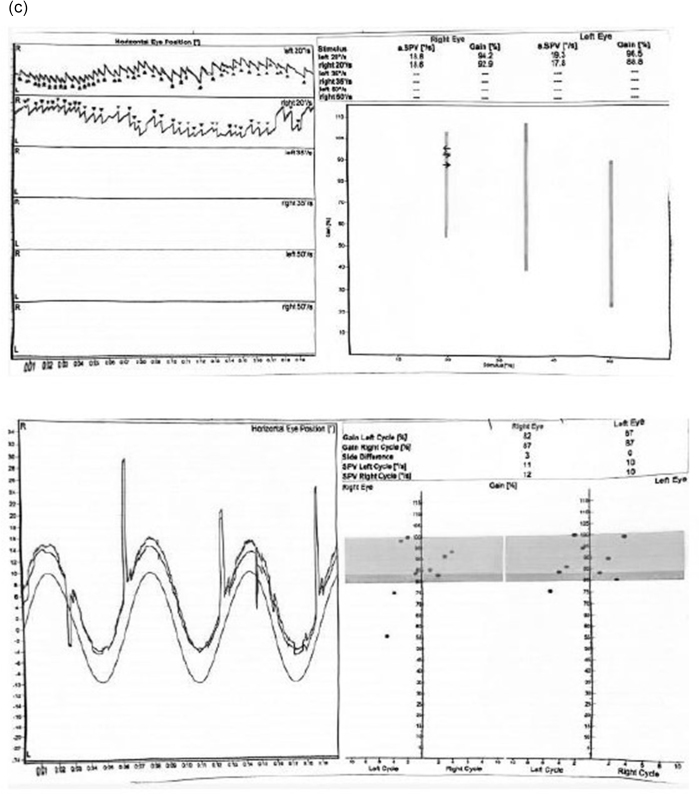
(a) Vestibular testing of the patient. (b) Vestibular testing of the patient. (c) Vestibular testing traces during horizontal and vertical conditions.

This figure presents the vestibular testing results of the patient, assessing equilibrium scores and various sensory and strategy analyses. The equilibrium score chart shows the patient’s performance across six different test conditions, with the composite score indicating an overall score of 62. The sensory analysis chart evaluates the patient’s reliance on somatosensory (SOM), visual, and vestibular inputs, showing that the patient’s performance is influenced more by SOM. The strategy analysis graph categorizes the patient’s dominant strategy for maintaining balance, showing a preference for ankle dominance over hip dominance. Finally, the center of gravity (COG) alignment graph indicates the patient’s postural stability during testing, with the patient’s COG alignment positioned near the ideal center during the trials. This testing ruled out significant vestibular dysfunction and helped confirm that the syncope episodes were not related to a vestibular disorder, pointing toward reflex-mediated causes, such as CSS.

This figure shows the horizontal and vertical traces of the patient’s vestibular testing over different time intervals. The horizontal and vertical tracings monitor the eye movements and vestibular responses in relation to the head movements during the test. In the traces, there are no abnormal oscillations or nystagmus, indicating that the vestibular system functions within normal limits, further ruling out a vestibular disorder as the cause of the patient’s syncope. The left and right traces reflect the symmetrical responses in both directions, confirming the normal function of the vestibulo-ocular reflex. These normal results support the diagnosis of reflex syncope as the likely cause of the patient’s recurrent episodes.

On physical examination, her pulse rate was 78 beats per minute; it was regular and of good character and volume. There were no radio-radial or radio-femoral delays. Her BP was recorded as 120/69 mmHg. Measurements were taken in supine, sitting, and standing, and no significant postural drop was recorded. The respiratory rate of the patient was 20 breaths per minute. Oxygen saturation was 97% on room air. The temperature was recorded as 37°C. Her weight was 63 kg, and her height was 154 cm.

Her general physical and systems examinations were normal. The apex beat was in the fifth intercostal space 4 cm from mid sternal line. The first and second heart sounds were normal and there were no audible murmurs or added heart sounds.

## Case management

### Investigations

An ECG was performed which showed sinus rhythm, heart rate of 72 per minute, normal electrical axis, and normal QT (Q wave and the T wave on an electrocardiogram) intervals. There was no intraventricular conduction abnormality, pathologic Q waves, preexcitation, ST segment changes, or T wave abnormalities. The patient-specific information was de-identified.

**Figure 3.**
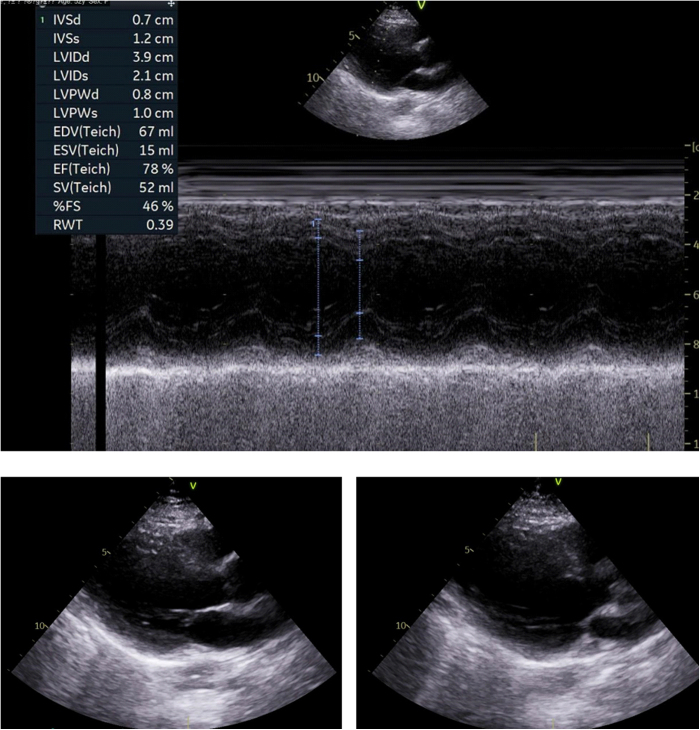
Echocardiogram results of the patient.

The echocardiogram showed an ejection fraction of 65%. Slides from her echocardiogram are shown in Figure 3. There was no ventricular hypertrophy left, cardiac chamber dilatation, regional wall motion abnormalities, or valvular heart diseases.

This figure shows the echocardiogram of the patient, providing a detailed assessment of cardiac function. The top panel displays the M-mode echocardiogram with measurements for key parameters:

- Interventricular septal thickness during diastole: 0.7 cm
- Left ventricular internal diameter during diastole: 3.9 cm
- Left ventricular posterior wall thickness during diastole: 1.0 cm
- Left ventricular posterior wall thickness during systole: 1.0 cm
- End-diastolic volume: 67 mL
- End-systolic volume: 15 mL
- Ejection fraction (EF): 72%
- Systolic wall thickening: 52 mL
- Systolic function score: 46.6%
- Relative wall thickness: 0.39

These parameters suggest normal left ventricular function, with an EF of 72%, which is within the normal range of 55%–70% for a healthy individual. The left ventricle appears to have normal wall thickness and no indication of hypertrophy.

The bottom panels show apical views of the left ventricle, providing further assessment of the heart’s structure and function. No regional wall motion abnormalities were observed, indicating that the left ventricle is functioning normally.

Overall, the echocardiogram suggests that the patient’s cardiac function is within normal limits, and there is no evidence of significant valvular heart disease, cardiac hypertrophy, or ventricular dilatation, ruling out major cardiovascular causes for the patient’s syncopal episodes.

CSM was performed while the patient was lying in bed and sitting upright. Both carotid arteries were auscultated for carotid bruit. Heart rate, BP, and ECG were continuously monitored throughout the procedure. While the face rotated contralaterally, CSM was performed by applying gentle and continuous pressure using the tips of the index, middle, and ring fingers at the site of maximum carotid artery pulsation between the anterior border of the sternocleidomastoid muscle and cricoid cartilage for 10 seconds. No asystole was recorded when CSM was performed during laying or sitting up. However, there was a fall in systolic BP during CSM performed on the right side while the patient was sitting upright in bed and the syncope was reproduced that lasted for about 7 seconds. The patient was assisted back to lying in bed, she was closely monitored, and she swiftly regained full consciousness with the return of normal heart rate and BP within approximately 4 seconds. The patient was kept under close surveillance.

## Patient’s clinical course and treatment adherence

The patient experienced recurrent syncope for 13 years, with episodes triggered by physical activity. After extensive testing, CSM confirmed vasodepressor-type CSS, leading to treatment with Fludrocortisone and lifestyle changes, including increased fluid intake. Over the course of 12 months, the patient reported no further episodes, indicating successful management. The patient adhered well to the treatment plan, and Fludrocortisone proved effective in preventing syncope, with normal vital signs during follow-up.

### Treatment and follow-up

The patient was educated in detail about her condition, its benign nature, and the risk of recurrence. She was educated about the triggers for her syncope. She was advised to increase fluid intake. Tablet Fludrocortisone 0.1 mg once daily was also advised. The patient was followed for 1 year, and there were no further episodes of syncope.

## Discussion

This case report presents a middle-aged woman diagnosed with vasodepressor-type CSS after experiencing recurrent syncopal episodes over a span of 13 years. Despite extensive diagnostic evaluations, including Holter monitoring, MRI, and EEG, the diagnosis remained unclear. CSM ultimately reproduced the syncope, confirming CSS as the underlying cause. The novel aspect of this case lies in the successful use of CSM to diagnose vasodepressor-type CSS, a subtype often overlooked in clinical practice due to its nonspecific nature. Additionally, the patient’s symptoms were effectively managed with lifestyle modifications and Fludrocortisone therapy, leading to a complete resolution of syncope after 1 year. This finding contributes to the growing body of evidence supporting noninvasive management for vasodepressor-type CSS.

CSS occurs due to an exaggerated response of the carotid sinus baroreceptors, leading to bradycardia or hypotension, resulting in syncope. The vasodepressor subtype of CSS is characterized by excessive vasodilation, causing significant drops in systolic BP. Treatment involves Fludrocortisone, which increases blood volume and BP, effectively preventing syncope episodes. This approach is supported by studies showing that Fludrocortisone reduces symptoms in vasodepressor-type CSS^[^7^]^.

The diagnosis of CSS, particularly the vasodepressor subtype, presents a diagnostic challenge. CSH, the precursor to CSS, is an age-related condition that has been underreported in the literature^[^7,12^]^. Many studies have primarily focused on the more dangerous forms of syncope, such as cardiac syncope, and less attention has been given to reflex syncope, especially CSS, in older adults. Previous research has demonstrated that CSM is the most effective diagnostic tool for CSS, as it directly triggers the syncope response and differentiates vasodepressor from cardioinhibitory types of syncope^[^4,14,15^]^. However, CSM is not routinely performed in clinical practice, despite its diagnostic accuracy. This case reinforces the value of CSM in confirming the diagnosis of vasodepressor-type CSS, a condition that is often overlooked due to its nonspecific clinical presentation.

Regarding treatment modalities, Fludrocortisone has been widely used to manage vasodepressor-type CSS by increasing fluid volume and improving BP^[^10,11^]^. Previous studies have shown Fludrocortisone to be effective in preventing syncope episodes in patients with CSS, particularly when used in conjunction with lifestyle modifications. This case aligns with the findings of positive outcomes with Fludrocortisone therapy in CSS management^[^16^]^. However, despite its widespread use, there remains limited data from studies evaluating the long-term effects of Fludrocortisone in managing CSS^[^17^]^. This case, while supporting the efficacy of Fludrocortisone, also highlights the need for more robust clinical trials to confirm its long-term benefits.

This study’s findings are consistent with existing literature that supports the role of CSM in diagnosing CSS, particularly in patients who do not respond to traditional diagnostic tests like ECG, MRI, and Holter monitoring. The use of Fludrocortisone for managing vasodepressor-type CSS also mirrors the results of several studies, which indicate that lifestyle modifications and Fludrocortisone are highly effective for treating this condition. However, the novelty of this case lies in the long-term follow-up (12 months), which demonstrates the sustained effectiveness of Fludrocortisone in preventing recurrent syncope, a finding that align with Fludrocortisone helps raise BP and improve symptoms in vasodepressor-type CSS^[^6,18^]^.

This case provides novel insights into the diagnosis and management of vasodepressor-type CSS. The successful use of CSM as a diagnostic tool, in combination with Fludrocortisone and lifestyle modifications, offers a promising approach to managing reflex syncope in older adults. The case also underscores the importance of considering CSS in the differential diagnosis of unexplained syncope, particularly when other diagnostic tests yield inconclusive results. Moreover, the long-term follow-up of the patient demonstrates that Fludrocortisone, when combined with lifestyle changes, can provide effective and sustainable symptom relief, offering a noninvasive alternative to more invasive procedures like pacemaker implantation.

Some studies, however, advocate for more invasive interventions such as pacemaker implantation in patients with cardioinhibitory-type CSS^[^19^]^. In contrast, this case supports the view that vasodepressor-type CSS can be effectively managed with conservative measures, thereby avoiding unnecessary invasive procedures. Vestibular testing, often employed to rule out neurologic causes of syncope, did not reveal any abnormalities in this case, further supporting the diagnosis of reflex syncope and reinforcing the diagnostic utility of CSM in distinguishing CSS from vestibular disorders.

The strengths of this case include the detailed diagnostic process, which highlights the importance of CSM in diagnosing vasodepressor-type CSS. The 12-month follow-up provides valuable evidence regarding the long-term effectiveness of Fludrocortisone in managing CSS, contributing to the literature by supporting the role of noninvasive treatments in reflex syncope management. Additionally, the case contributes to the understanding of CSS in the context of older adults, a group often underrepresented in clinical studies.

However, the limitations of this study include the single-case design, which limits the ability to generalize the findings to a broader population. While the findings are consistent with existing literature, the lack of randomized controlled trials and comparative studies on the efficacy of Fludrocortisone in managing CSS remains a significant gap in the evidence base. Larger studies with more diverse patient populations are needed to confirm the findings and provide stronger evidence for the long-term management of CSS.

## Conclusion

This case underscores the diagnostic value of CSM in patients with recurrent syncope, especially in the absence of clear underlying cardiovascular conditions. The use of Fludrocortisone in the vasodepressor-type CSS is an effective and noninvasive treatment option, and this report adds to the growing body of evidence supporting conservative management for this condition.

## Data Availability

The data are available from the corresponding author upon reasonable request.
